# Staff radiation doses during Sentinel Lymph Node procedure of breast cancer from injection to surgeon

**DOI:** 10.1016/j.heliyon.2024.e30706

**Published:** 2024-05-04

**Authors:** Fayzan Ahmed, Majid Iqbal, Syed Mansoor Naqvi, Javaid Iqbal

**Affiliations:** a Department of Physics, NED University of Engineering & Technology, Karachi, Pakistan; b Department of Physics, Federal Urdu University of Arts, Science & Technology, Gulshan-e-Iqbal Campus, Karachi, Pakistan; c Department of Radiology, Aga Khan University, Karachi, Pakistan; d Department of Radiology, Nuclear Medicine Section, Liaquat National Hospital (LNH), Karachi, Pakistan; e Department of Radiology, Nuclear Medicine Section, Karachi Institute of Radiotherapy and Nuclear Medicine (KIRAN), Karachi, Pakistan

**Keywords:** Sentinel Lymph Node (SLN) Biopsy (SLNB), Radiation protection, Nuclear medicine (NM), Scintigraphy, Effective dose, Dose rate, Cumulative dose, Optically Stimulated Luminescence dosimeter (OSLD), Active dosimeter, Passive dosimeter

## Abstract

The Sentinel Lymph Node (SLN) or Sentinel Lymph Node Biopsy (SLNB) technique involves various professionals from different departments in clinical settings to manage breast cancer patients properly. Tracing the nodular involvement of breast cancer patients requires radiation source Tc^**99m**^ labeled with colloidal albumin to be injected at the tumor site. The patient becomes a radiation source for a sufficient time, which concerns the Nuclear Medicine (NM) and surgical staff. The study aims to provide the radiation doses of staff in the NM department during the SLN scintigraphy procedure and obtain an empirical model for calculating the radiation doses to staff in the surgical department from that particular patient. Radiation doses in SLN technique for breast cancer patients are minimal, and a sufficient number of SLN biopsy procedures can be performed by hospital staff within the category of non-radiation workers.

## 1 Introduction

Sentinel node (SN) biopsy identifies the most significant prognostic factors in breast cancer, melanoma, and other solid tumors. It has become a standard procedure in managing breast cancer and melanoma patients [[1], [2], [3]]. The sentinel lymph node (SLN) is the primary lymph node of the tumor's lymphatic drainage. The presence of tumor infiltration of SLN is provided by the SLN biopsy.

Only a 10 % non-palpable breast carcinoma rate and a 30 % positive rate for palpable breast carcinomas are observed in the axillary lymph node dissections. It is also observed that 70–90 % of axillary lymph node dissections are extraneous [4]. Hematoma, ipsilateral arm oedema, neuropathy, and infections are complications of axillary lymph node dissection [5,6]. Thus, the SLN biopsy technique can effectively prevent unnecessary surgery complications and related issues.

With suitable tracers introduced into the surrounding tissue and appropriate detection technology, these drainage channels can be identified and mapped conveniently. Generally, this mapping is accomplished by administering radioactive substance labeled with colloidal material with particles that are within a suitable size range. The radioactive isotope technicium-99 m (Tc^**99m**^) is a gamma ray-emitting radionuclide with a short half-life labeled with this colloidal material. This enables the acquisition of images for the distribution of labeled colloidal pharmaceutical by utilization of a conventional gamma camera [7] having large-field-of-view (LFOV) equipped with low-energy (ultra-) high-resolution collimators [8] and verification for node dissection the surgical staff uses gamma probe. Blue dye tracers may also be used with or without a radioactive tracer, which allows for the visualization of drainage paths.

This study aims to assess the radiation exposure levels of medical staff during Sentinel Lymph Node (SLN) scintigraphy procedures in the Nuclear Medicine (NM) department. Additionally, the study attempts to create an empirical model to predict radiation doses for surgical department staff assisting breast cancer patients during surgery or biopsy. Although the SLN scintigraphy procedure involves low radioactivity levels (about 1 mCi), staff members are nonetheless exposed to radiation. Studying the radiation doses that hospital workers are engaged in SLN procedures could aid in creating guidelines to reduce staff radiation exposure.

## 2 Materials and methods

**Fig. 1:**
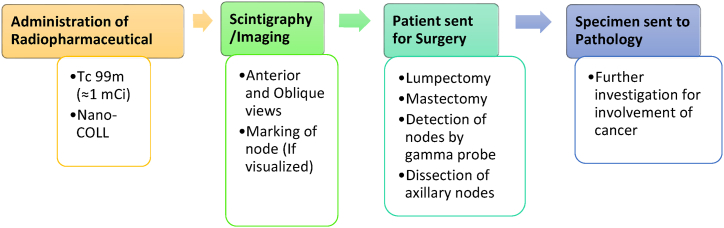
SLN biopsy procedure flow chart.

The study was performed at the Nuclear Medicine Department, Liaquat National Hospital (LNH) Karachi. The study presented here is divided into two parts. First, it aimed to provide the radiation doses of the staff of NM involved in SLN radioactive tracer administration and marking nodes visualized by a gamma camera. Secondly, the formation of an empirical model to predict the doses to staff engaged during the SLN tracer stage in nuclear medicine or radiology and in operating theatre (OT) where surgeons and assistants are present. A flow chart representation is shown in Fig. 1.

### 2.1 SLN procedure and instrumentation

The tracer is injected in four quadrants of 0.5 cc volume (4th part of activity/0.5 cc) around the tumor site intradermally as standard practice. The SLN procedure involves a Tc^**99m**^-colloid tracer (Nanocoll, GE Healthcare Limited, UK) as a radioactive tracer. The radioactive isotope in this radiopharmaceutical is Technicium Tc^**99m**^, which has a photopeak of gamma-ray emission of 140.5 keV [9], and its physical life is 6 h [10].

For two months of routine work in the nuclear medicine section, the staff of radiology and nuclear medicine involved in SLN Tc^**99m**^-colloid tracer and nodal marking were given separate radiation monitoring badges: Optically Stimulated Luminescence Dosimeter (OSLDs) InLight® LANDAUER Inc specifically for the duration of this particular procedure. Along with OSLD, an active portable dosimeter (FS2011+, Graiger, China) was also provided during the patient interaction within the NM department. The OSLDs were calibrated and read from LANDAUER Inc. systems present at Aga Khan University Hospital (AKUH) Karachi; moreover, the portable dosimeter FS 2011+ was calibrated from the Pakistan Institute of Nuclear Science and Technology (PINSTECH). The FS 2011+ can also act as survey meters, so measurements at various distances were taken. So, the OSLDs serve as passive dosimeters while the FS 2011+ is an active dosimetre. Both OSLD and portable dosimeters served the purpose of calculating the cumulative dose of the NM staff.

### 2.2 Population studied

Staff radiation doses were monitored for all sentinel lymph node (SLN) patients during the two-month research period. 33 individuals had the SLN diagnostic process throughout this period. All patients were female and mainly from the urban population of Sindh province of Pakistan. There were no unique criteria for patient selection for this study, as all patients underwent the same level of radioactivity and imaging method. Consequently, staff involvement remained uniform across all patients undergoing SLN procedures.

### 2.3 Statistical analysis

The doses measured by the OSLD are taken as cumulative doses. The NM workers wore similar OSLD badges during this study; this was an additional badge provided to staff. The active dosimetry was given as NM staff came in contact with the radiation source while administering radiopharmaceuticals and the patient's positioning and nodal marking. The radiation doses were noted for each activity and presented as cumulative doses.

To estimate radiation dose rates from the Tc^**99m**^ source, the active dosimeter measured dose rates at various distances, from which average dose rates were measured along with the standard deviation of measured dose rates.

### 2.4 Mathematical modelling

As per European Association of Nuclear Medicine (EANM) guidelines, Bluemel, Christina et al., 2015 [11] and departmental practices usually 1–1.10 mCi is injected, so a 1.05 mCi Tc^**99m**^ Normal Saline solution is taken and a dose rate by the portable dosimeter was measured at various distances from the radiation source. The response of portable dosimeters was recorded and used for mathematical modeling to estimate the doses received by staff in NM during SLN scintigraphy and the surgical team during nodal dissection and surgery of breast cancer patients. The model is based on the radioactive decay equation applied to the decay of Tc^**99m**^.

The mathematical model for the estimation of effective dose to staff is

(1) $$D={\dot{D}}^{\prime }\times Decay\;factor\times t$$

The relationship of absorbed or effective dose with radioactivity is given by

$$\dot{D}\;\alpha \;A$$

Here A represents the activity of the source and $\dot{D}$ Is the absorbed dose rate (Gray/Hour, Gy/hr) and the portable dosimeters provide effective dose rate readings in units of Sievert/Hour (Sv/hr, mSv/hr or μSv/hr). t is the time spent near the patient.

For taking different values of activity, the proportional relation gives

$$\frac{\dot{D}}{{\dot{D}}_{ref}}=\frac{A_{inj}}{A_{ref}}$$

(2) $${\dot{D}}^{\prime }=\left(\frac{A_{inj}}{A_{ref}}\right){\dot{D}}_{ref}$$

where $A_{ref}$ represents the activity of the source at which dose rates ${\dot{D}}_{ref}$ were measured by portable ddosimeter $A_{inj}$ is the activity injected for which the dose rate ${\dot{D}}^{\prime }$ is estimated.

The radioactive decay equation accounts for the reduction in the activity of the radioactive source.

(3) $$Decay\;factor=e^{-\lambda T}$$

where Decayfactor gives the reduction in activity with respect to T time between injection and procedure for which staff interact with the patient. λ is the decay constant related to the source's half-life.

By using equations (2), (3) equation (1) becomes

(4) $$D=\left\{\left(\frac{A_{inj}}{A_{ref}}\right){\dot{D}}_{ref}\right\}\times e^{-\lambda T}\times t$$

Equation (4) measures the effective dose to the staff by providing appropriate parameters. All the calculations were performed using MS Excel®.

**Fig. 2:**
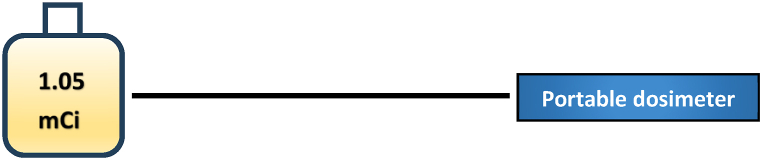
Measurement by portable at various distances from Tc^99^m source.

The dose rate measurement was obtained by a portable dosimeter at various distances. The pictorial representation of the measurement scenario is represented in Fig. 2.

## 3 Results

The measurements obtained are represented in tabular form. Table 1 gives the effective dose estimation of the NM staff involved in the SLN procedure Tc^**99m**^-colloid labeled injection and nodal marking during scintigraphy. The measurements are obtained by OSLD (Passive dosimeter). These are separate OSLDs other than staff uses in their routine work. The number of patients per year is calculated based on the radiation worker's permissible dose (Category A), i.e., 20 mSv/year [12].

**Table 1:** Effective doses measured by OSLD (Passive dosimeter).

No of Patients	Cumulative dose (mSv)	Estimated Dose per patient (mSv)	No of Patients per year (Estimated)
33	0.0850	0.0026	7765

Table 2 gives the values of adequate doses of NM staff by portable dosimeter's cumulative dose display specific to interaction tasks with either radioactive source or injected patient.

**Table 2:** Effective doses measured by portable dosimeter (Active dosimeter).

No of Patients	Task	Cumulative dose (mSv)
33	Administration of Activity	0.046
	Patient Positioning and Marking	0.035

The effective dose is obtained by two dosimeters with separate systems and measurement methods. The estimated dose per patient by OSLD and portable dosimeter is approximately 0.0026 mSv and 0.0024 mSv, respectively. The obtained measurements can give the number of patients according to annual dose limits recommended by ICRP [13], which are 1 mSv/year or 1000 μSv (Category B) for the general public and 20 mSv/year or 20,000 μSv (Category A) for radiation workers. The allowed number of patients per year is obtained by considering only the SLN procedure, and the annual dose of radiation worker will reach a maximum, i.e., 20 mSv.

As for the other part of this study, the values in Table 3 are utilized as reference values for the mathematical model, and the cumulative doses for staff during surgery are estimated. The response of portable dosimeter varies at a specific distance, so the standard deviation was also measured.

**Table 3:** Dose rate measured by portable dosimeter at various distances from source (Active dosimeter).

Distance *Cm*	Average dose rate μSv/hr	Standard deviation
Close	142.69	0.55
5	49.28	0.49
10	25.18	0.15
15	14.61	0.09
20	9.93	0.08
30	5.46	0.11
40	3.11	0.04
50	2.24	0.07
60	1.63	0.26
80	0.93	0.16
100	0.63	0.03
120	0.51	0.03
140	0.54	0.09
170	0.24	0.02
200	0.21	0.01

**Fig. 3:**
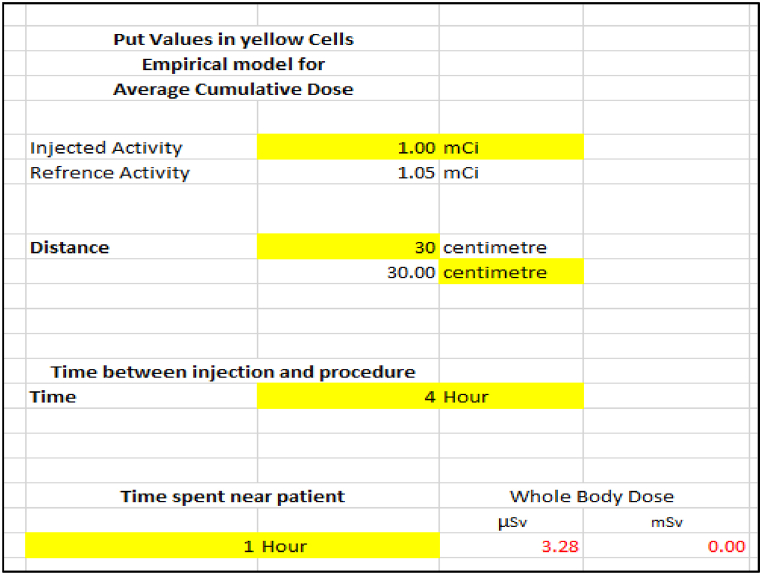
SLN staff doses model. Kimura, Fuyo et al., 2015 [14].

The mathematical model formed by using MS Excel® is shown in Fig. 3 with an illustrative example. The parameters, such as injected activity, distance from the patient, the time between injection and procedure, and time spent near the patient, will provide an estimated dose for the staff.

## 4 Discussion

Currently, the use of radioisotope and blue dye for sentinel lymph node biopsy (SLNB) for axillary staging in breast cancer is every day [15]. SLN dissection has proved to be effective and highly accurate with a low false negative rate [16], but simultaneously, the procedure poses radiation risks to the staff involved at every stage. It contains the radioactive source from radioactive tracer administration to the pathology department, where the patient and related specimen moves.

As the radiative substance injected is of few mCi, it may not account for large exposure values, which makes the classification of other staff as radiation workers (category A). The measurement of radiation doses to the surgical and pathological team is attempted in many studies, some of them given in Table 4 [4,[14], [17], [18]], where the analyses were performed by measuring the personal monitoring dosimeters of surgical staff using Thermoluminescent dosimeter (TLDs) and survey meters. Recording the specific time duration and distance from the patient in the operation theatre provided means of calculating staff doses by dose rates of survey meter observations provided in Table 3 and the mathematical model formed in this study, as shown in Fig. 3.

**Table 4:** Comparison between previous studies and the model.

Studies	Injected Activity mCi	Distance between staff (surgeon) and patient cm	The time between injection and surgery Hour	Time spent near the patient Hour	Average dose μSv	Average dose by model μSv
1. De Kanter, A. Y. et al., 2003 [17]	0.81	30	4	2	8.2	7.31
2. Klausen, T. L. et al., 2005 [18]	1.35	20	2	1	10	10.14
3. Bekiş, Recep et al., 2009 [4]	0.8–1.2	50	3	1.66	3.35	3.52
4. Kimura, Fuyo et al., 2015^a^ [14]	1	30	4	1	3	3.28

a *No exact information on parameters was given, so average values were taken*.

**Fig. 4:**
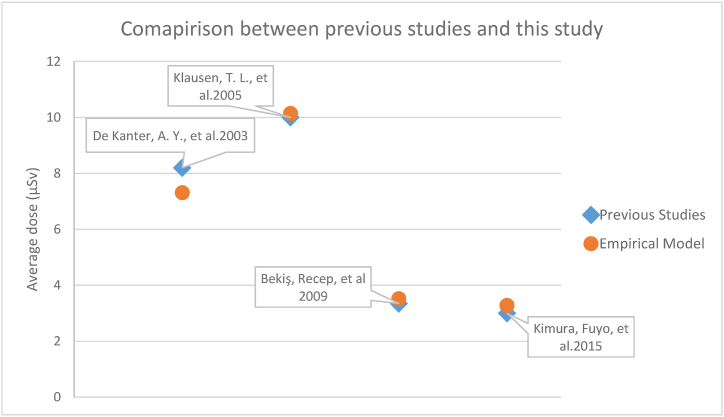
Comparison between previous studies and model.

In our study, the doses of NM staff were measured for this specific procedure, which provides evidence that the radiation doses are minimal per patient. The radiation protection measures are well-defined and reflect good medical practice in our center. The unsealed Tc^**99m**^ source observed dose rates at various distances, which are applicable for NM and surgical staff in OT in routine procedure practices. The mathematical model, made on simple equations, provides radiation doses of staff comparable to the previous studies, as shown in Fig. 4. These values can be refined by considering various factors such as scattering, dosimeter response, detection efficiency, energy response of the detector, and other factors.

From a radiation protection point of view, surgical staff involved in these procedures are non-radiation workers. Based on previous studies, surgeons can perform 305–630 procedures annually within the classification of non-radiation workers.

Although the procedure is very safe for all staff as it involves a minimal amount of radioactive isotopes [19], it is imperative to implement additional measures. These measures should adhere to the ALARA principle (As Low As Reasonably Achievable). The radiation doses to surgical staff can be minimized by following universal precautions such as wearing gloves to avoid radioactive contamination, using long forceps, properly planning to reduce the overall surgical procedure time, and separating containers for dissection specimens. Such precautions will reduce the time of exposure and contamination issues, reducing radiation exposure significantly. Lead protective clothing is not an essential requirement and may restrict staff mobility.

The staff members in the NM department are already familiar with the regulations governing radiation protection. They are recognized as radiation workers and have completed the necessary radiation protection training. The radiation exposure of additional assisting or tertiary care workers who may be present in the NM department to support the patient and any family members will also be limited due to the low activity injected for the SLN procedure. Further reducing their radiation exposure is that these people typically do not spend long periods near the patient being injected. Other centers may use such methodology to estimate the radiation doses for various steps involved in the SLN procedure and recommend NM staff and staff members present at the surgery or OT room.

While impactful, the study has a few limitations that should be acknowledged. The main emphasis of this study is to estimate radiation doses for SLNB in the context of breast cancer staging, biopsy, and surgery. Nevertheless, it is essential to note that the emphasis on a particular operation may not comprehensively capture the wide range of NM tests, hence constraining the applicability of the results to other NM procedures. Furthermore, the study's utilization of approximations for procedures in the OT may introduce uncertainties in dose estimation, given that practices can vary among institutions and individuals. Despite these limitations, the study offers valuable information on radiation protection measures and dose calculations for SLNB. This emphasizes the need for additional research to comprehend radiation doses to departmental and tertiary care staff involved in various other NM procedures.

## 5 Conclusion

Measuring radiation doses to staff is crucial in setting up the radiation protection program; however, the measurements can be performed in many ways. The procedures in which staff is involved with radiation sources and radioactive patients are of concern. Such a methodology is provided in our study as well. This study stands significant as such analysis of radiation doses to staff during the SLN procedure in the context of radiation protection has yet to be reported from Pakistan. Our study also provides a simple approach for estimating the average radiation dose to NM and surgical staff by properly providing distance from the patient and time spent near the patient. The mathematical model-based approach can be used to estimate the doses for each staff member. These various methods of staff dosimetry provide prominent evidence that radiation doses in SLN technique for breast cancer patients are very minimal and a sufficient number of SLN biopsy procedures can be performed by hospital staff within the category of non-radiation workers; however, keeping the radiation protection cardinal rules in account and proper planning will help to reduce these doses and alleviate the radiation-related concerns of individuals.

## Ethical statement

There are no ethical issues with this study. Informed consent was obtained from all the participants of this study.

## Funding statement

The research did not receive any speciﬁc grant from funding agencies in the public, commercial, or non-profit sector.

## Data availability statement

All related data utilized in this study is provided in the manuscript. The mathematical model formed and any other additional data will be available to readers upon request.

## CRediT authorship contribution statement

**Fayzan Ahmed:** Writing – review & editing, Writing – original draft, Visualization, Software, Methodology, Investigation, Formal analysis, Data curation. **Majid Iqbal:** Writing – review & editing, Writing – original draft, Visualization, Software, Methodology, Investigation, Formal analysis, Data curation. **Syed Mansoor Naqvi:** Writing – original draft, Visualization, Supervision, Methodology, Conceptualization. **Javaid Iqbal:** Validation, Supervision, Methodology, Conceptualization.

## Declaration of competing interest

The authors declare that they have no known competing financial interests or personal relationships that could have appeared to influence the work reported in this paper.
